# Learning Compact Multispectral Signatures for Geographical-Origin Authentication of *Pinellia ternata* via Correlation-Guided Deep Modeling

**DOI:** 10.3390/molecules31173138

**Published:** 2026-09-07

**Authors:** Zhihui Fan, Shaowen Jing, Chao Ma, Sen Wang, Zhenzhen Chen, Jiayu Huang, Mingkun Zhang

**Affiliations:** 1 College of Information Engineering, Henan University of Science and Technology, Luoyang 471023, China; fzh@haust.edu.cn (Z.F.);; 2 Artificial Intelligence Department, Yuxin Electronic Technology Group Co., Ltd., Zhengzhou 450000, China; wangsen@eathink.com.cn (S.W.);; 3 College of Electronics and Information Engineering, South China University of Technology, Guangzhou 510641, China

**Keywords:** *Pinellia ternata*, multispectral imaging, geographical origin, Pearson correlation, feature selection, multilayer perceptron, nested group validation, medicinal plant authentication

## Abstract

Geographical authentication of medicinal plant materials remains challenging because multispectral variables are often highly collinear and sample grouping can complicate reliable model validation. Existing correlation-based feature-selection strategies also require careful adaptation to multiclass problems to avoid artificial ordering of class labels and information leakage during model development. Therefore, this study aimed to develop a compact and leakage-controlled multispectral learning framework for geographical-origin discrimination. This study analyzed 800 physical *Pinellia ternata* samples from Gansu Xihe, Sichuan Neijiang, Sichuan Chengdu, and Chongqing Dianjiang (200 samples per origin). Each physical sample was represented by 31 mean grayscale intensities calculated from Otsu-segmented multispectral regions of interest. A Pearson-correlation-guided deep multilayer perceptron (PCG-DeepMLP) was constructed by estimating one-vs-rest band relevance and inter-band redundancy only within the training data. The key methodological innovation is a unified multiclass-aware, relevance–redundancy spectral-learning framework in which class-specific one-vs-rest Pearson relevance is coupled with inter-band redundancy control and embedded within leakage-controlled grouped model development. By learning the spectral subset exclusively from each training partition before nonlinear classification, the framework produces compact and complementary multispectral signatures while preserving multiclass structure and strict independence of held-out groups. Model and feature-selection settings were chosen by three-fold grouped cross-validation within each training partition. PCG-DeepMLP retained 9–21 bands and achieved the highest mean accuracy (0.9812 ± 0.0135), macro-F1 (0.9812 ± 0.0135), Matthews correlation coefficient (MCC; 0.9752 ± 0.0179), and macro-AUC (0.9994 ± 0.0006) among seven models. Its macro-F1 was higher than that of 1D-CNN, 1D-ResNet, full-band MLP, PLS-DA, and random forest after Holm correction. Performance was estimated through a strict nested group-wise internal validation scheme, with every outer test fold remaining isolated from feature selection, preprocessing, and model optimization. These findings demonstrate that multiclass-aware relevance–redundancy learning can retain complementary *Pinellia ternata* origin-discriminative information in a compact and stable spectral representation, enabling accurate geographical-origin authentication while providing a principled basis for reduced-channel acquisition and future independent multi-batch validation.

## 1. Introduction

Pinelliae Rhizoma, the dried tuber of *Pinellia ternata* (Thunb.) Breit., is a chemically complex medicinal material whose quality control is complicated by species substitution, phenotypic heterogeneity, and variation in metabolite abundance. Recent untargeted and spatial metabolomic studies have documented marked chemical differences between authentic Pinelliae Rhizoma and adulterants, and among plant phenotypes [1,2]. These findings support objective analytical authentication rather than reliance on morphology alone.

Pinelliae Rhizoma is an important plant-derived material with both medicinal and health-related applications. Pinelliae Rhizoma has a long history of medicinal use in China, Japan, Korea, and Southeast Asian countries [3]. Recent evidence places global demand for *P. ternata* raw materials at approximately 7000 t [3,4]. This substantial demand makes reliable provenance control particularly important because geographical origin is associated with measurable phytochemical variation. A metabolomic comparison of *P. ternata* from two producing regions identified 573 metabolites and 155 differential metabolites, with clear multivariate separation and systematic differences in major metabolite classes [5]. More recently, UHPLC-MS/MS analysis of samples from Gansu, Hebei, and Hubei revealed significant geographical variation in anti-inflammatory cyclodipeptides and indicated that origin should be considered in quality assessment and the consistency of medicinal effects [6]. These findings provide direct evidence that geographical provenance is a quality-relevant attribute of *P. ternata* and strengthen the practical need for rapid and objective origin authentication to support provenance verification, raw-material quality control, and standardized utilization. Modern pharmacological studies have reported multiple biological activities associated with *P. ternata*, including antitussive and expectorant, antiemetic, anti-inflammatory, and anti-asthmatic effects [3,7]. These pharmacological properties, together with its broad utilization and the sensitivity of botanical materials to cultivation environment, processing, and post-harvest conditions, increase the importance of consistent raw-material quality and reliable provenance control. Consequently, rapid and objective geographical-origin discrimination is important for quality assurance, authenticity evaluation, and standardized utilization of Pinelliae Rhizoma.

Geographical origin can influence the composition and commercial value of plant-derived materials through differences in genotype, soil, climate, cultivation, harvest, and post-harvest handling. Contemporary traceability studies combine chemical fingerprints, chromaticity, electronic sensing, elemental profiles, nuclear magnetic resonance, or fluorescence spectroscopy with multivariate classification [8,9,10]. In general, these origin-authentication approaches are constructed through a common analytical sequence: samples with known provenance are first characterized using chemical, spectral, elemental, or sensor-response measurements; the resulting multivariate fingerprints are then preprocessed and represented by informative variables; finally, a supervised classification model is trained to associate these analytical signatures with predefined geographical classes [8,10]. Once established, the model assigns an unknown sample to the most compatible origin according to its measured fingerprint, so the reliability of the final decision depends jointly on the analytical signal, feature representation, and validation strategy. Similar authenticity problems have been examined for honey and other food matrices, where analytical fingerprints are valuable only when sampling design and validation reflect the intended use [11].

Visible and near-infrared methods are attractive because they are fast, non-destructive, and compatible with compact instruments. Portable NIR spectroscopy has recently discriminated the origins of food-medicine homologous materials, while conventional NIR and mid-infrared data have supported robust discrimination in other heterogeneous matrices [12,13]. Machine-learning-assisted UV/Vis-NIR analysis has also been used for turmeric adulteration detection [14]. A recent *Molecules* study further emphasized the need to connect herbal-medicine classification to interpretable spectral evidence [15].

Recent Vis/NIR studies have combined origin discrimination, mold detection, and limited-band authentication for food-derived botanical materials [16,17,18]. They also illustrate that band-aware image acquisition and compact neural architectures are relevant methodological considerations for deployable optical systems [19,20]. Related work on counterfeit citrus materials and multispectral origin identification further supports the use of deep models in non-destructive botanical-material analysis [21,22].

Hyperspectral and multispectral imaging extend spectroscopy by associating spectral response with an imaged region. Current applications include fruit and vegetable quality assessment [23,24]. Medicinal-material authenticity analysis provides a second important application [25,26]. Plant-organ and industrial-scale assessments further illustrate the breadth of this approach [27,28]. Deep architectures can learn nonlinear spectral relationships, as demonstrated in rice variety identification, ginseng analysis, and black tea fermentation monitoring [29,30,31]. Nevertheless, when the predictor dimension is modest and samples are grouped by acquisition or source, a tuned classical method may outperform a neural network; this possibility should be tested rather than assumed.

For *P. ternata*, available studies have largely focused on chemical characterization, authenticity assessment, and phenotype-related compositional differences rather than compact multispectral geographical-origin discrimination [1,2]. Related origin-authentication studies on other botanical materials demonstrate the potential of spectral and multispectral sensing, but their direct transfer to *P. ternata* is not straightforward because low-dimensional multispectral variables can remain strongly collinear, informative bands may be redundant, and multiclass labels cannot be treated as naturally ordered numerical targets [12,16,22]. In addition, feature selection performed before or outside grouped model validation can introduce information leakage and overestimate generalization. Therefore, a specific methodological gap remains for *P. ternata*: a compact origin-discrimination framework is needed that simultaneously handles multiclass relevance, inter-band redundancy, and grouped leakage-controlled validation.

**Figure 1:**
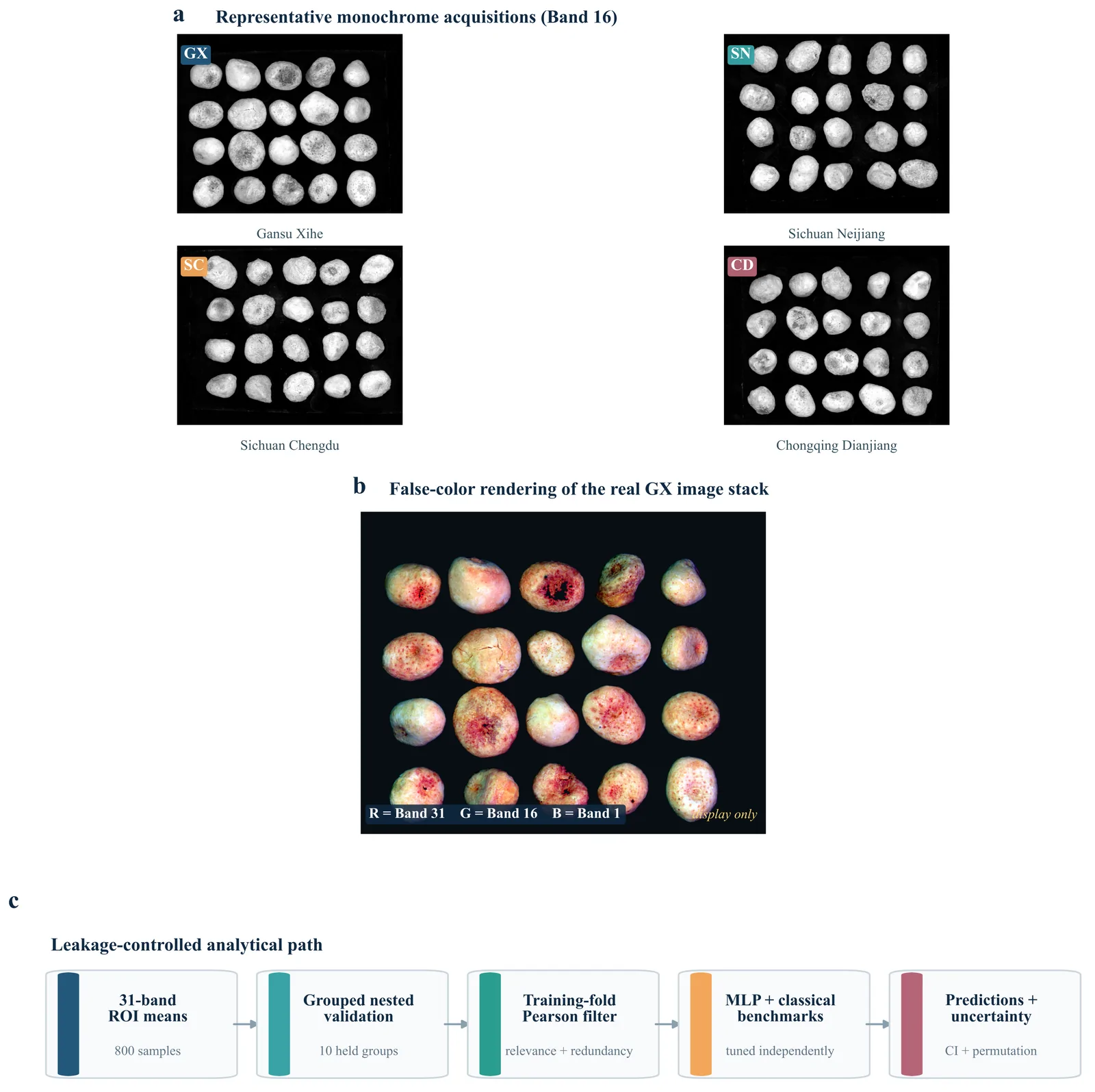
Multispectral image examples and analytical workflow. (**a**) Representative Band 16 monochrome acquisitions for the four origins. A group-4 tray not used in any other manuscript figure was selected for each origin, and each tray contained 20 physical samples. (**b**) Display-only false-color rendering of the Gansu Xihe group-4 tray, obtained by mapping individually normalized Bands 31, 16, and 1 to red, green, and blue, respectively; this rendering was not used for modeling. (**c**) Data-processing and model-validation workflow, including extraction of 31-band ROI mean features, nested group-wise validation, training-fold Pearson-correlation-based assessment of one-vs-rest band relevance and inter-band redundancy, model optimization and benchmarking, and evaluation of held-out predictions and uncertainty. CI, confidence interval; MLP, multilayer perceptron; ROI, region of interest.

Pearson filtering is computationally transparent, but correlating each band with integer-coded multiclass labels would impose an artificial ordering on the origins. We therefore define class-wise Pearson relevance against one-vs-rest indicator variables and combine it with inter-band redundancy control. Both operations are learned inside training folds. This design is particularly appropriate for the present multiclass and highly collinear multispectral ROI data. Direct Pearson correlation with integer-coded origin labels would create an artificial ordinal relationship among classes, whereas one-vs-rest indicators provide class-specific relevance without assuming such an order. Moreover, because strongly correlated bands can carry largely overlapping information, relevance ranking alone may retain redundant variables; the additional inter-band correlation constraint therefore favors a more compact set of complementary spectral features. Performing both operations exclusively within the training partitions further prevents feature-selection information from entering the held-out data. Pearson correlation is not the only possible variable-selection strategy, but its transparent relevance measure, low computational burden, and straightforward integration with redundancy control make it well suited to the present 31-band grouped dataset. The study objectives were to (i) characterize the 31-band ROI-intensity structure of *P. ternata* from four Chinese origins, (ii) evaluate a Pearson-correlation-guided deep multilayer perceptron (PCG-DeepMLP) under nested group-aware validation, (iii) compare it with full-band MLP, 1D-CNN, 1D-ResNet, PLS-DA, RBF-SVM, and random forest models, and (iv) quantify band-selection stability and the limits of the available dataset. Representative source acquisitions, a display-only false-color rendering, and the analytical sequence are integrated in Figure 1.

## 2. Results and Discussion

### 2.1. Dataset Integrity and Spectral Structure

The supplied table contained 800 unique sample identifiers, 31 numeric band features, and one origin label. There were no missing cells, duplicated rows, or duplicated sample identifiers. Each origin contributed 200 samples, and each numbered origin group contributed 20 samples. ROI mean grayscale intensity ranged from 15.568 to 237.574. These pre-modeling checks are summarized in Table 1.

**Figure 2:**
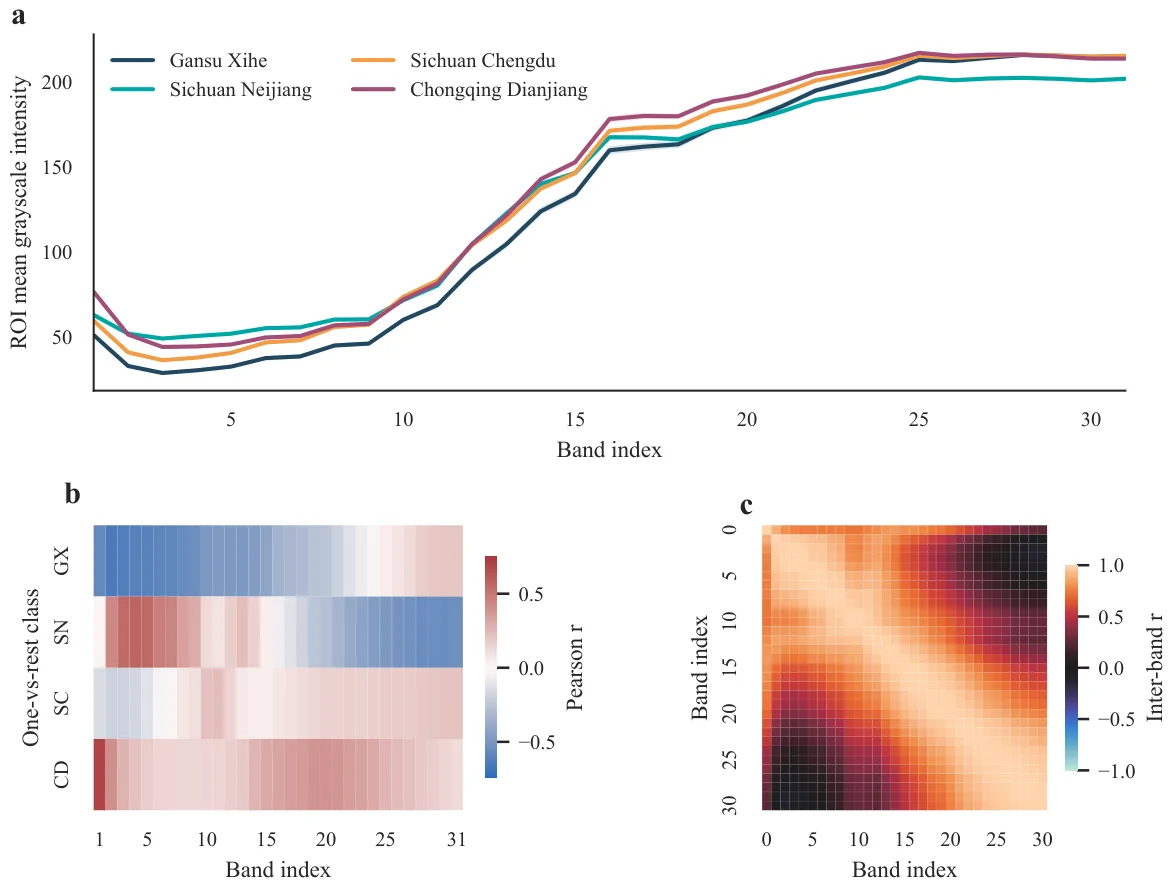
Spectral-intensity structure of the dataset. (**a**) Origin-specific mean ROI grayscale intensity across Bands 1–31; shaded 95% confidence intervals are narrow and are partly obscured by the mean curves. (**b**) One-vs-rest Pearson correlation between each band and each origin indicator. (**c**) Inter-band Pearson correlation matrix. GX, Gansu Xihe; SN, Sichuan Neijiang; SC, Sichuan Chengdu; CD, Chongqing Dianjiang.

The class-average profiles followed a shared intensity trajectory but differed systematically in amplitude and local shape (Figure 2a). One-vs-rest Pearson correlations changed sign and magnitude across both bands and classes (Figure 2b), demonstrating why a single correlation with integer-coded class labels would be inappropriate. The inter-band matrix showed extensive collinearity, particularly among adjacent and late-index bands (Figure 2c), supporting redundancy-aware selection.

### 2.2. Latent-Space Separation

**Figure 3:**
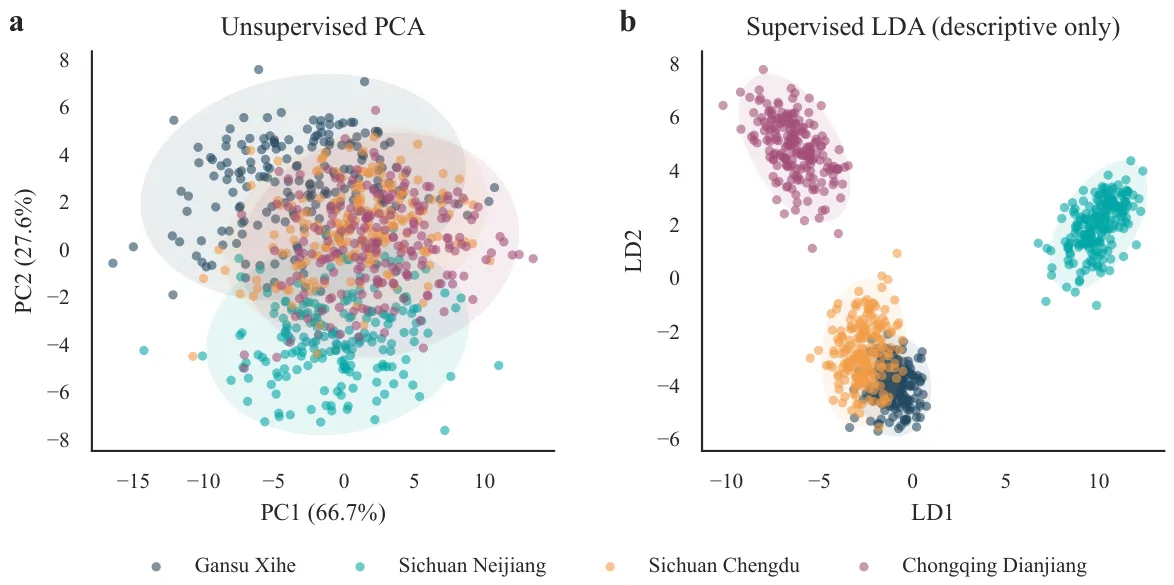
Descriptive latent-space visualization. (**a**) Unsupervised PCA scores with 95% covariance ellipses. (**b**) Supervised LDA scores fitted to the complete dataset for visualization only. Predictive performance was not calculated from these coordinates.

The first two principal components explained 94.3% of standardized feature variance (PC1, 66.7%; PC2, 27.6%), yet the four origins overlapped substantially in the unsupervised score space. By contrast, supervised LDA produced pronounced class separation, with the largest residual overlap between Gansu Xihe and Sichuan Chengdu (Figure 3). The LDA panel provides a descriptive complement to the nested outer-fold performance analysis.

### 2.3. Nested Group-Aware Model Comparison

**Figure 4:**
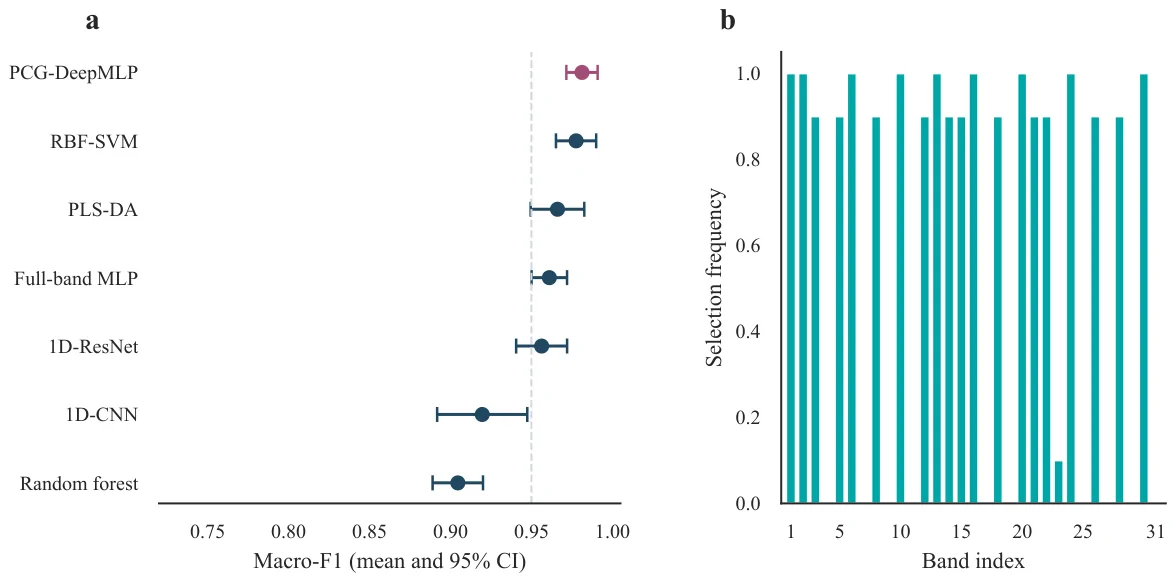
Comparative performance and band-selection stability. (**a**) Macro-F1 with 95% t-based confidence intervals across 10 outer folds. (**b**) Proportion of outer folds in which each band was retained by PCG-DeepMLP. Feature selection was repeated within each training fold.

Model ranking was consistent across accuracy, macro-F1, MCC, and macro-AUC (Table 2; Figure 4a). PCG-DeepMLP provided the highest mean accuracy (0.9813 ± 0.0135) and macro-F1 (0.9812 ± 0.0135), followed by RBF-SVM (accuracy, 0.9775 ± 0.0175; macro-F1, 0.9776 ± 0.0174). The full-spectrum 1D-ResNet (0.9563 ± 0.0220) and 1D-CNN (0.9196 ± 0.0389) were included as independent deep baselines; PLS-DA, the full-band MLP, and random forest yielded macro-F1 values of 0.9661 ± 0.0231, 0.9611 ± 0.0153, and 0.9045 ± 0.0217, respectively.

Paired Wilcoxon tests across the 10 outer folds, with Holm correction, showed that PCG-DeepMLP outperformed 1D-CNN (median macro-F1 difference, 0.0452; adjusted *p* = 0.0117), 1D-ResNet (0.0130; adjusted *p* = 0.0433), random forest (0.0768; adjusted *p* = 0.0117), the full-band MLP (0.0247; adjusted *p* = 0.0320), and PLS-DA (0.0125; adjusted *p* = 0.0495). Across the seven-model benchmark, the correlation-guided deep model ranked first for accuracy, macro-F1, MCC, and macro-AUC.

### 2.4. PCG-DeepMLP Discrimination and Stable Spectral Evidence

PCG-DeepMLP realized subsets of 9–21 bands, depending on the outer training partition. Bands 1, 2, 6, 10, 13, 16, 20, 24, and 30 were retained in all 10 folds (Figure 4b; Table 3). According to the wavelength mapping of the multispectral system, these consistently retained bands correspond to nominal central wavelengths of 365, 380, 420, 465, 520, 585, 680, 770, and 940 nm, respectively. Their recurrence across all 10 outer folds indicates high selection stability under the present grouped-validation framework. The selected wavelengths span the ultraviolet, visible, and near-infrared regions rather than forming a single contiguous spectral interval, suggesting that discrimination is supported by complementary information distributed across different portions of the measured spectrum. The selected subset therefore emphasized both early and late portions of the band sequence rather than a single contiguous region.

Fold-wise realized subset sizes, selected hidden architectures, regularization values, and redundancy thresholds are reported in Table 4. Both the input subset and the neural configuration varied within the bounded inner search, with all choices made only from each outer training partition.

**Figure 5:**
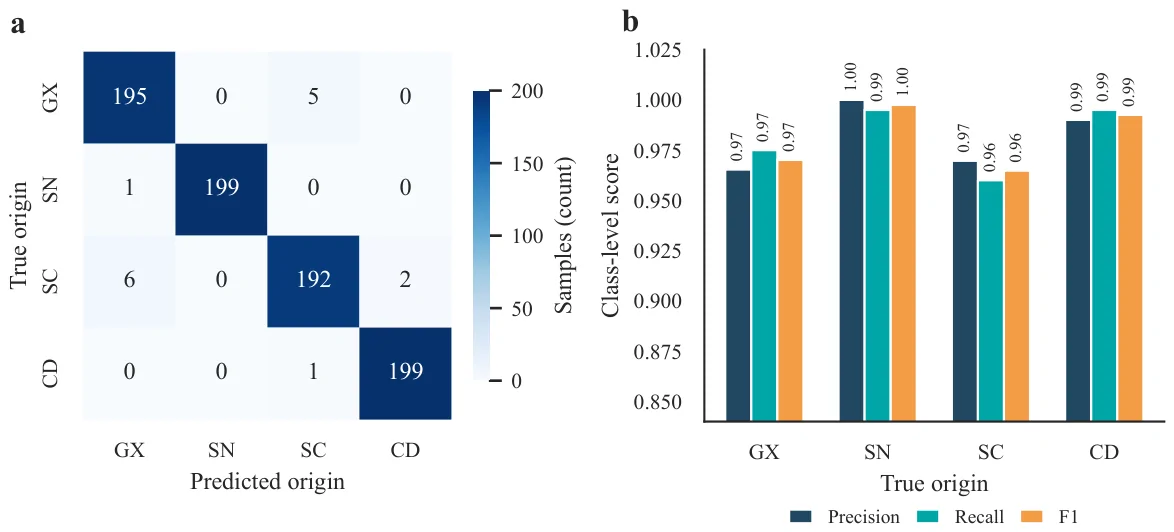
Out-of-fold PCG-DeepMLP diagnostic performance. (**a**) Confusion matrix in physical-sample counts. (**b**) Class-specific precision, recall, and F1 score. GX, Gansu Xihe; SN, Sichuan Neijiang; SC, Sichuan Chengdu; CD, Chongqing Dianjiang.

Aggregated out-of-fold predictions correctly classified 195 of 200 Gansu Xihe samples, 199 of 200 Sichuan Neijiang samples, 192 of 200 Sichuan Chengdu samples, and 199 of 200 Chongqing Dianjiang samples. Gansu Xihe and Sichuan Chengdu were the principal confusion pair: five Gansu Xihe samples were assigned to Sichuan Chengdu and six Sichuan Chengdu samples were assigned to Gansu Xihe. Class-specific recall ranged from 0.960 to 0.995 (Figure 5b), and the aggregated out-of-fold macro-AUC was 0.9987.

### 2.5. Robustness Checks

**Figure 6:**
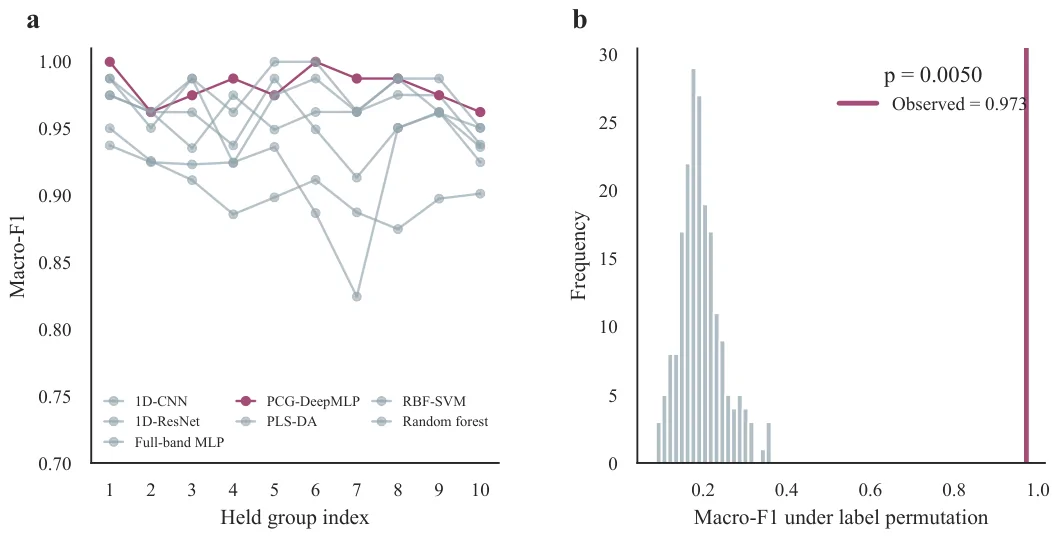
Robustness analyses. (**a**) Outer-fold macro-F1 profiles for all models; PCG-DeepMLP is highlighted. (**b**) Block-level label-permutation distribution for a fixed PCG-SVM pipeline (200 permutations). The vertical line denotes the observed grouped-cross-validation score.

PCG-DeepMLP macro-F1 ranged from 0.9625 to 1.0000 across held group indices, indicating stable performance across the encoded group structure (Figure 6a). A separate 200-permutation block-label test was performed with a computationally tractable PCG-SVM pipeline under the same 10 held groups (Figure 6b). Its observed macro-F1 was 0.9725, whereas the null mean was 0.1953 ± 0.0519; the finite-permutation *p*-value was 0.00498, confirming a strong non-random multivariate origin signal.

### 2.6. Out-of-Fold Decision Confidence

**Figure 7:**
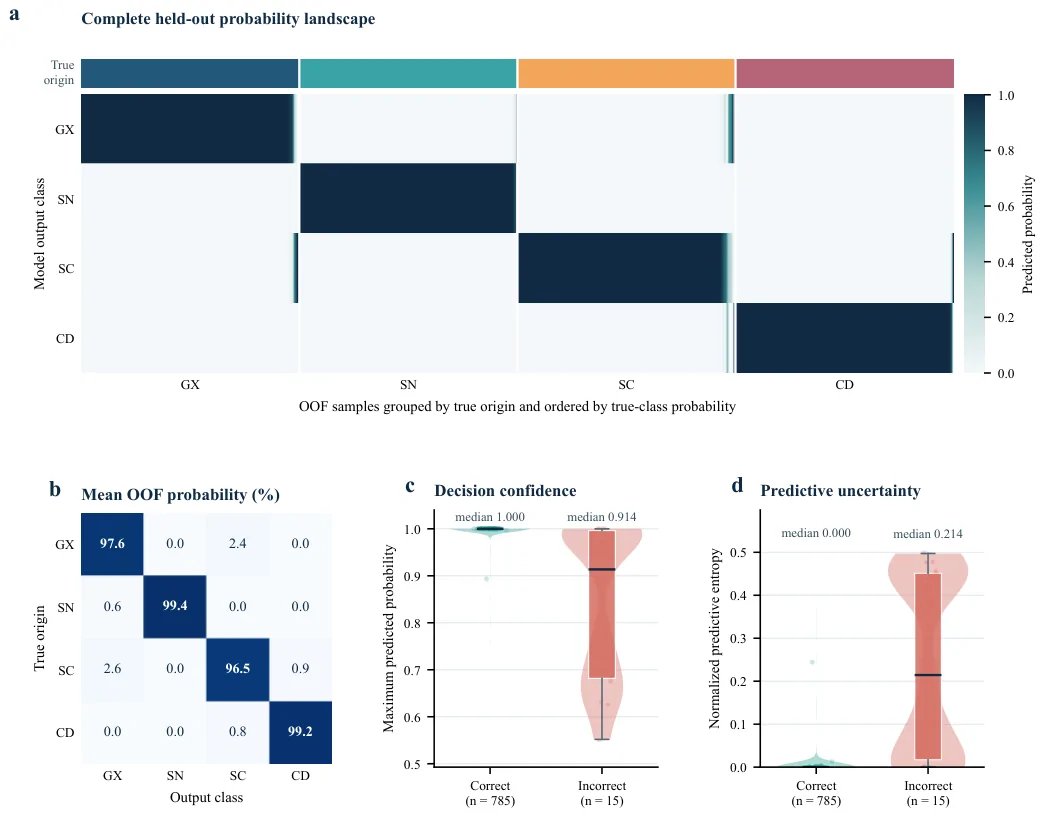
Decision-level structure of PCG-DeepMLP out-of-fold probabilities. (**a**) Probability heatmap for all 800 samples, ordered within each true-origin block by descending true-class probability; the upper strip denotes true origin. (**b**) Mean predicted-probability matrix by true origin. (**c**) Distribution of maximum predicted probability for correct and incorrect classifications. (**d**) Distribution of normalized predictive entropy. Box centers and limits denote the median and interquartile range; violins show kernel-density estimates. Each probability vector was generated only by the outer model for which that sample was held out. GX, Gansu Xihe; SN, Sichuan Neijiang; SC, Sichuan Chengdu; CD, Chongqing Dianjiang.

Inspection of all 800 held-out probability vectors showed that correct classifications had a median maximum probability of 1.000, compared with 0.914 for errors. Median normalized predictive entropy was 0.000 for correct cases and 0.214 for errors (Figure 7). The mean probability matrix localized the principal ambiguity to the Gansu Xihe–Sichuan Chengdu pair, providing a decision-level view of the model’s discrimination structure.

### 2.7. Origin Information in Multispectral ROI Means

The data support a strong association between ROI grayscale profiles and declared geographical origin. Comparable studies have shown that origin, processing, and adulteration can alter spectral or chemical fingerprints in Chinese medicinal materials and citrus-derived products [16,32,33]. Chemometric authentication of root and rhizome herbs by ATR-FTIR and hyperspectral analysis of Atractylodis Rhizoma further demonstrates that complex plant matrices can retain origin- or identity-related multivariate signals [34,35].

The present profiles should not be interpreted as direct compound measurements. Visible and near-infrared contrast can reflect interacting effects of pigmentation, water, scattering, morphology, and organic constituents. Recent work on citrus profiles, pomelo Vis-NIR measurements, and a broad review of pigment-oriented hyperspectral imaging illustrates both the chemical sensitivity and the non-specificity of optical bands [36,37,38]. Direct metabolite assays or calibrated reflectance measurements would be required to link individual *P. ternata* bands to defined compounds.

### 2.8. Group-Aware Validation Matters

The identifiers encoded 10 numbered groups within each origin. Their experimental meaning was not supplied, but samples sharing such an index may be closer in acquisition order, material source, or handling. Holding out the same number across all four origins created balanced 80-sample outer tests and reduced the risk that group-specific structure appeared in both training and testing. Similar concerns arise in small-sample hyperspectral deep learning, where random sample-level splits can overstate generalization if technical or biological replicates are separated [39].

The grouped split evaluates all models across balanced 80-sample outer tests and provides a direct basis for benchmarking their performance across the encoded groups. Multi-batch and multi-instrument extensions can further broaden the traceability domain, as illustrated by citrus, tea, and ginseng applications [40,41,42]. It is also important to distinguish grouped internal validation from fully independent deployment evaluation. Although each outer test partition was completely excluded from feature selection, preprocessing, hyperparameter optimization, and model fitting, all 800 samples were acquired within the same overall experimental dataset. Accordingly, the macro-F1 of 0.9812 characterizes the discrimination capability of PCG-DeepMLP under the present controlled acquisition conditions and group-wise validation framework. Broader generalization can be further assessed using independently collected production batches spanning different acquisition periods, suppliers, instruments, and operating environments. Such evaluation will provide an additional basis for determining the robustness and transferability of the proposed framework in practical geographical-origin authentication.

### 2.9. Pearson Guidance, Compactness, and Model Choice

The PCG procedure avoids two common sources of bias. First, one-vs-rest indicators prevent the numerical labels 1-4 from imposing a fictitious geographic order. Second, feature ranking and redundancy filtering are repeated inside each training fold, preventing selection leakage. Stable recurrence of nine bands across all folds suggests reproducible relevance within this dataset, while fold-dependent inclusion of other bands reflects strong inter-band correlation.

The PCG-DeepMLP used 9-21 inputs rather than all 31, with architectures ranging from 32-16 to 128-64-32 selected only within the outer training partitions. It delivered the highest mean grouped-validation metrics and significantly exceeded the independently tuned full-spectrum 1D-CNN and 1D-ResNet baselines, PLS-DA, the full-band MLP, and random forest after Holm correction. The result demonstrates the value of integrating class-wise Pearson relevance, redundancy control, and nonlinear learning for compact multispectral origin traceability. Deep networks have been successful when richer spatial-spectral representations or larger hyperspectral sequences are available, as in attention-based ginseng, tobacco, and mandarin models [40,43,44].

Several factors may explain the high discrimination performance observed in the present study. First, the four origins exhibited systematic differences in both the amplitude and local shape of their multispectral profiles, indicating that the 31-band measurements contained substantial origin-associated information. At the same time, the strong inter-band collinearity meant that many variables carried overlapping information; the Pearson-guided relevance-redundancy procedure therefore concentrated the input representation on a smaller set of complementary bands before nonlinear classification. Second, the comparatively lower performance of the 1D-CNN and 1D-ResNet can be related to the structure of the available data. Each physical sample was represented by a short sequence of only 31 band-wise ROI means, without pixel-level spatial or texture information. Under this compact representation, the local receptive-field and hierarchical feature-extraction advantages of convolutional architectures are less pronounced than they are for high-dimensional hyperspectral cubes or longer spectral sequences. In contrast, the MLP can directly model nonlinear interactions among selected wavelengths, including informative bands that are not spectrally adjacent. The similarly strong performance of RBF-SVM further suggests that the present task is dominated by a compact nonlinear spectral-discrimination structure rather than by the need for progressively deeper convolutional feature extraction.

Possible acquisition- or batch-related effects were also considered when interpreting the high performance. The group-wise validation scheme withheld complete acquisition trays from model development, so samples from the same tray were not divided between the corresponding training and test partitions, thereby reducing the possibility that simple tray-specific characteristics directly inflated the reported performance. Nevertheless, geographical origin was not independently crossed with all potentially influential factors, such as production source, harvest conditions, storage history, and acquisition period. Consequently, origin-associated spectral variation may contain contributions from these covarying factors in addition to geographical effects themselves. The present results therefore demonstrate strong and reproducible discrimination of the declared origins under the current acquisition design, while independently collected multi-batch and multi-period datasets will allow geographical effects to be separated more rigorously from broader production and acquisition variability.

### 2.10. Practical Implications and Dataset Context

The stable band subset could inform a reduced-channel acquisition design, but wavelength identities are essential before hardware translation. Recent wavelength-selection and data-fusion studies in chrysanthemum and tea demonstrate how selected wavelengths can guide simplified sensors when the wavelength calibration is explicit [41,45]. Here, reporting only Bands 1-31 is the scientifically defensible choice.

The present dataset records 31 Otsu-ROI mean grayscale features for 800 physical samples and retains the original image stacks for provenance. The revised acquisition description provides the wavelength mapping, wavelength-specific exposure procedure, and dark- and white-reference radiometric calibration used for the multispectral measurements. However, sampling year, harvest stage, supplier, voucher information, and storage history were not independently balanced across the four geographical origins. These variables may influence optical response through differences in material composition, moisture status, morphology, processing, and post-harvest condition. Because they were not orthogonally crossed with geographical origin, the present design cannot completely partition the observed spectral variation into purely geographical effects and covarying production- or sample-history effects. Accordingly, the measured differences are interpreted as origin-associated spectral signatures under the present sampling framework rather than as causal markers of geographical location alone. The group-wise acquisition-tray validation reduces direct acquisition-level information leakage, but it does not remove these potential upstream confounding effects. Independent multi-year, multi-source, and multi-batch sampling will therefore be important for further separating geographical effects from broader production and handling variability.

The availability of explicit wavelength mapping improves the spectral interpretability of the selected variables; however, wavelength-level stability should be distinguished from compound-specific interpretation. The measured responses of plant-derived samples can reflect combined effects of absorption, scattering, pigmentation, moisture, morphology, and multiple organic constituents. Therefore, the consistently selected wavelengths are interpreted here as reproducible predictive spectral features rather than direct markers of individual metabolites. Complementary chemical measurements and independently acquired multispectral datasets will be valuable for determining the physicochemical basis and cross-dataset stability of these wavelengths.

The predictive metrics should also be interpreted according to the scope of geographical traceability. Accuracy, macro-F1, and AUC quantify how consistently the measured multispectral signatures discriminate the four declared origins under the present validation design, whereas overall traceability is a broader system-level capability and does not increase linearly with classification performance. Comprehensive traceability additionally involves reliable provenance records, batch continuity, robustness to acquisition and environmental variation, and verification across independently collected materials. Therefore, the present results support the analytical geographical-origin discrimination component of a broader traceability framework rather than equating predictive accuracy directly with the overall level of traceability.

## 3. Materials and Methods

### 3.1. Samples and Labels

The analyzed dataset comprised 800 physical *P. ternata* samples. Numerical labels denoted Gansu Xihe (1), Sichuan Neijiang (2), Sichuan Chengdu (3), and Chongqing Dianjiang (4), with 200 samples per origin. Each geographical origin comprised 200 physical samples. During image acquisition, 20 samples were placed on each sample tray, resulting in ten acquisition trays per origin. The tray identifier was retained only to define the group-wise data partitions used during validation and was not included as a predictor or geographical class label. Each ROI represented one physical sample. Accordingly, the analytical input matrix contained 800 rows and 31 numerical predictor columns, corresponding to 800 physical samples and 31 multispectral ROI-mean features, respectively. The geographical-origin label and numbered group identifier were stored separately and were used only for supervised classification and group-aware data partitioning; neither was included in the predictor matrix.

### 3.2. Multispectral Imaging Platform

The acquisition configuration matched the self-built platform. The system comprised a multi-wavelength LED illumination module, integrating sphere, CCD camera, focusing unit, imaging lens, sample stage, supporting frame, and computer control. The multispectral illumination module contained 31 discrete bands with nominal central wavelengths of 365, 380, 390, 400, 410, 420, 430, 440, 455, 465, 475, 500, 520, 535, 570, 585, 595, 620, 655, 680, 700, 730, 750, 770, 805, 820, 850, 880, 900, 940, and 980 nm, corresponding sequentially to Bands 1–31. The design full width at half maximum (FWHM) was 10 nm for the 365–400 nm channels, 14 nm for the 410–730 nm channels, and 20 nm for the 750–980 nm channels. The CCD specification reported for this platform was 1600 × 1200 pixels with 12-bit depth. The integrating sphere provided diffuse illumination to reduce spatial non-uniformity.

Before sample acquisition, the exposure time was optimized independently for each wavelength to ensure adequate signal intensity while avoiding detector saturation, and the determined exposure setting was then kept unchanged for the corresponding band during reference and sample imaging. Dark-reference images were acquired under completely blocked-light conditions using the opaque black lens cap supplied with the Lumenera camera. White-reference images were acquired from an in-house diffuse reference panel prepared by uniformly coating a flat substrate with high-purity BaSO_4_, and the spectral reflectance characteristics of the reference panel were verified using a spectrometer before calibration. For each band, radiometric correction was performed using the corresponding sample, dark-reference, and white-reference images according to R=(I−D)/(W−D), where *I* denotes the raw sample-image intensity, *D* denotes the dark-reference intensity, *W* denotes the white-reference intensity, and *R* denotes the corrected relative reflectance. This band-wise correction compensates for camera dark response as well as wavelength-dependent variations in illumination intensity and detector response. The corrected multispectral images were subsequently used for sample-background segmentation and ROI determination, after which the mean valid-pixel response within each ROI was calculated independently for the 31 bands to construct the spectral feature vector for each physical sample.

### 3.3. ROI Segmentation and Feature Table

Before the present analysis, Otsu thresholding had been applied to separate each physical sample from its background, and the mean grayscale intensity within the resulting ROI had been calculated independently for each band. Thus, every row contained a 31-dimensional vector of ROI means. The supplied values ranged from 15.568 to 237.574, consistent with an 8-bit-scaled analytical table. Original tray images were available for provenance and for the display-only false-color rendering in Figure 1.

### 3.4. Pearson-Correlation-Guided Feature Selection

Let x_ij denote the ROI mean intensity of sample i at band j, and let y_i be its declared origin. Within each training partition, a one-vs-rest indicator was formed separately for every class c; no integer coding of origin labels was used in the correlation calculation:

(1) $$z_{i}^{\left(c\right)}=I\left(y_{i}=c\right),\;c\in \left\{1,2,3,4\right\}.$$

For each band and class, Pearson relevance was calculated from the training observations only. The multiclass relevance score was the largest absolute class-wise association:

(2) $$r_{j,c}=\frac{\mathrm{cov}(x_{j},z^{\left(c\right)})}{\mathrm{sd}(x_{j})\mathrm{sd}(z^{\left(c\right)})},\;R_{j}=\max_{c}\left|r_{j,c}\right|.$$

Bands were ranked by decreasing R_j. For candidate maximum subset size K and redundancy threshold tau, the greedy selector retained the next ranked band only when its absolute Pearson correlation with every retained band was below tau:

(3) $$S\left(K,\tau \right)=\left\{j_{1},\ldots ,j_{m}\right\},\;m\leq K,\;\left|\mathrm{corr}(x_{j},x_{l})\right|<\tau \;\forall \;j\neq l\in S.$$

The selected subset and classifier configuration were chosen entirely within the inner grouped folds by maximizing mean macro-F1; g denotes an inner validation fold, and theta denotes the classifier settings:

(4) $$\left(K^{*},\tau^{*},\theta^{*}\right)=\underset{K,\tau ,\theta }{\arg\;\max}\;\frac{1}{G}\sum_{g=1}^{G}{\mathrm{MacroF1}}_{g}.$$

Candidate values were K in {12, 16, 20, 24} and tau in {0.95, 0.98, 0.995, 1.01}. Because redundancy sometimes stopped selection early, the realized outer-fold subsets contained 9–21 bands. The fitted selector, standardizer, and classifier were then refit on the complete outer training partition before a held-out group index was predicted.

### 3.5. Classification Models

PCG-DeepMLP combined training-fold Pearson selection, standardization, and an MLPClassifier with an L-BFGS optimizer, a four-class output, and a maximum of 1200 iterations. Its bounded candidate space included maximum selected-band counts of 12, 16, 20, and 24; redundancy thresholds of 0.95, 0.98, 0.995, and 1.01; hidden-layer structures of 32-16, 64-32, 96-48, 128-64, and 128-64-32; ReLU or tanh activation; and L2 alpha values from 10^−6^ to 10^−2^. The realized selected subset could be smaller than K after redundancy control.

Two full-spectrum PyTorch neural baselines were also evaluated on the ordered 31-band sequence: a 1D-CNN with two convolutional blocks (width 16 or 24, batch normalization, GELU, adaptive average pooling, and dropout) and a 1D-ResNet with a convolutional stem and two residual blocks (width 12 or 16, batch normalization, GELU, and dropout). For each architecture, three predefined optimization configurations were compared within the inner folds; models were trained for 120 epochs with AdamW and a batch size of 96 after training-fold standardization. Remaining comparators were a full-band MLP, RBF-SVM, PLS-DA, and random forest. The full-band MLP hidden structures tested were 64-32 and 64-32-16; RBF-SVM used C in {1, 10, 100} and gamma in {scale, 0.01, 0.1}; PLS-DA used 2, 4, 6, or 8 latent components; random forest used 600 trees, max_features in {square root, 0.7}, and minimum leaf sizes of 1 or 2. Class balancing was applied to SVM and random forest.

### 3.6. Validation and Statistical Analysis

Primary performance was estimated using 10 outer folds. In outer fold *g*, the corresponding acquisition tray from each of the four origins was held out, giving 80 test samples in total (20 samples per origin), while the remaining 720 samples were used for training and model selection. Hyperparameters were selected by three-fold GroupKFold cross-validation within the 720-sample training partition using macro-F1. For PCG-DeepMLP, 40 deterministically seeded settings were sampled from the stated bounded candidate space in each outer fold. For each 1D neural baseline, one of three predefined configurations was selected in the same inner grouped folds. All preprocessing and Pearson selection were refitted for each candidate and fold.

Reported fold metrics were accuracy, balanced accuracy, macro precision, macro recall, macro-F1, MCC, and macro one-vs-rest ROC AUC. Means, standard deviations, and 95% t-based confidence intervals were calculated across the 10 outer folds. Model comparisons used paired two-sided Wilcoxon signed-rank tests on outer-fold macro-F1 with Holm adjustment. Given the limited number of paired outer folds, these inferential comparisons were treated as supportive evidence; small non-significant differences between closely performing models were interpreted descriptively rather than as evidence of statistical superiority. A 200-permutation signal test was performed at the 20-sample origin-group block level using a fixed PCG-SVM pipeline for computational tractability; *p* was calculated as (1 + number of null scores at least as large as observed)/(1 + number of permutations). Two-dimensional PCA and LDA fitted to the full dataset were used only for descriptive visualization. Analyses were executed in Python 3.13 with NumPy, pandas, SciPy, scikit-learn, PyTorch, Matplotlib, and seaborn.

## 4. Conclusions

The present study establishes a clear link between multispectral characteristics and geographical-origin discrimination of *P. ternata*. The 31-band ROI profiles revealed reproducible origin-associated spectral differences, while the recurrent selection of wavelengths spanning the ultraviolet, visible, and near-infrared regions indicates that discrimination relied on complementary information distributed across the measured spectrum. The results further demonstrate that combining multiclass-aware Pearson relevance, inter-band redundancy control, and nonlinear learning provides a compact and stable representation for geographical-origin discrimination under group-aware validation. A remaining limitation is that the present evidence was obtained from a single experimental dataset, and broader generalization across independently collected batches, acquisition periods, and instruments requires further evaluation. Future work will therefore focus on independent multi-batch and cross-instrument validation, together with physicochemical interpretation of the selected wavelengths. Within the present dataset and grouped-validation framework, PCG-DeepMLP therefore provides an effective analytical approach for geographical-origin discrimination, while its stable band subset provides a basis for subsequent reduced-channel acquisition design. The high predictive performance supports the origin-authentication component of traceability, whereas comprehensive traceability additionally depends on provenance continuity, independent validation, and robustness across production and acquisition conditions.

## Figures and Tables

**Table 1:** Dataset characteristics and pre-modeling quality checks.

Characteristic	Value
Physical samples	800
Origins	4 (200 per origin)
Band features	31
Numbered groups	10 per origin; 20 samples per origin-group
Observed grayscale range	15.568–237.574

**Table 2:** Nested group-wise performance (mean ± standard deviation across 10 outer folds). AUC was calculated one-vs-rest with macro averaging.

Model	Accuracy	Macro-F1	MCC	Macro-AUC
PCG-DeepMLP	0.9812 ± 0.0135	0.9812 ± 0.0135	0.9752 ± 0.0179	0.9994 ± 0.0006
RBF-SVM	0.9775 ± 0.0175	0.9776 ± 0.0174	0.9707 ± 0.0226	0.9990 ± 0.0012
PLS-DA	0.9662 ± 0.0229	0.9661 ± 0.0231	0.9557 ± 0.0297	0.9938 ± 0.0065
Full-band MLP	0.9613 ± 0.0150	0.9611 ± 0.0153	0.9491 ± 0.0190	0.9977 ± 0.0019
1D-ResNet	0.9563 ± 0.0222	0.9563 ± 0.0220	0.9427 ± 0.0296	0.9957 ± 0.0052
1D-CNN	0.9200 ± 0.0387	0.9196 ± 0.0389	0.8950 ± 0.0506	0.9861 ± 0.0073
Random forest	0.9050 ± 0.0214	0.9045 ± 0.0217	0.8743 ± 0.0283	0.9790 ± 0.0067

**Table 3:** Ten most stable PCG-DeepMLP band features. Relevance is the maximum absolute one-vs-rest Pearson correlation estimated in the corresponding training fold.

Band	Folds Selected	Mean Rank	Mean Relevance
1	10	1.00	0.700
2	10	2.00	0.650
6	10	4.80	0.587
30	10	6.60	0.549
13	10	9.80	0.486
10	10	10.30	0.483
24	10	13.00	0.458
20	10	16.00	0.382
16	10	19.00	0.342
3	9	3.00	0.630

**Table 4:** Fold-wise PCG-DeepMLP settings selected exclusively within each outer training partition and the resulting held-group macro-F1. K/selected gives the maximum candidate K and the realized selected-band count.

Held Group	K/Selected	Hidden Layers	Activation	L2 Alpha	Threshold Tau	Macro-F1
1	24/21	128-64-32	tanh	$1\times 10^{-6}$	0.995	1.0000
2	20/20	128-64	tanh	$1\times 10^{-2}$	0.995	0.9625
3	20/20	32-16	relu	$1\times 10^{-5}$	0.995	0.9750
4	20/20	96-48	tanh	$1\times 10^{-2}$	0.995	0.9875
5	24/20	64-32	tanh	$1\times 10^{-2}$	0.995	0.9750
6	24/20	128-64	tanh	$1\times 10^{-3}$	0.995	1.0000
7	20/20	128-64-32	tanh	$1\times 10^{-2}$	0.995	0.9875
8	12/9	128-64-32	relu	$1\times 10^{-2}$	0.95	0.9875
9	24/20	32-16	relu	$1\times 10^{-2}$	0.995	0.9750
10	20/20	128-64-32	tanh	$1\times 10^{-3}$	0.995	0.9625

## Data Availability

The data presented in this study are not publicly available at present because they were generated using shared laboratory equipment and are subject to laboratory data-management restrictions. The dataset will be made publicly available after the restrictions are lifted.

## Abbreviations

The following abbreviations are used in this manuscript:

AUC	Area under the receiver operating characteristic curve
LDA	Linear discriminant analysis
MCC	Matthews correlation coefficient
MLP	Multilayer perceptron
PCG	Pearson-correlation-guided
PLS-DA	Partial least squares discriminant analysis
ROI	Region of interest
SVM	Support vector machine
